# Effects of underground coal mining disturbance on bacterial community diversity and its assembly processes

**DOI:** 10.1371/journal.pone.0322014

**Published:** 2025-05-07

**Authors:** Fei Wang, Zehao Zhang, Zhaohua Lu, Junting Guo, Zhanyong Fu, Jingkuan Sun

**Affiliations:** 1 School of Chemical & Environmental Engineering, China University of Mining and Technology (Beijing), Beijing, China; 2 State Key Laboratory of Water Resource Protection and Utilization in Coal Mining, Beijing, China; 3 Shandong Key Laboratory of Eco-Environmental Science for the Yellow River Delta, Shandong University of Aeronautics, Binzhou, China; University of Udine: Universita degli Studi di Udine, ITALY

## Abstract

Vegetation restoration is an important indicator for evaluating the health of mining ecosystems. Soil microorganisms influence soil material cycling and play an important role in vegetation restoration. Clarifying the effects of mining on soil microbial communities has spleen benefits for vegetation restoration. In this study, we analyzed the effects of mine disturbance on soil bacterial communities by high-throughput sequencing and setting up sampling sites at different distances from the mine site. The results showed that the α-diversity of the bacterial community gradually increased and the β-diversity changed with increasing distance from the mine site. The changes in β-diversity were mainly from rare taxa. Distance increased the relative abundance of Actinobacteria, Acidobacteria, and Chloroflexi, but significantly decreased the relative abundance of Proteobacteria and Firmicutes. Bacterial networks within the mine had higher robustness and edge counts, and the robustness of molecular ecological networks decreased with increasing distance. To adapt to the nutrient-poor nature of mine soils, microbial populations preferred to cooperate with each other. The bacterial community assembly processes in all sample sites were dominated by random dispersal and heterogeneous selection, contributing 49.24% and 43.87%, respectively. The bacterial community assembly processes in the mining area were dominated by deterministic processes (heterogeneous selection, 69.4%). Salt, SUE and SOM had the greatest impact on total, rich and rare bacterial community α-diversity, which were significantly negatively, positively and positively correlated, respectively. TK, SOM, SUE and SALP had significant effects on the bacterial community structure of all three taxa. βNTI increased significantly with increasing differences in TN and ALSP and decreased with increasing differences in salt. The above results suggest that mining affects the diversity, composition, and assembling processes of bacterial communities, and that the effect on abundant and rare taxa are different.

## 1. Introduction

Mining is the main method of coal production adopted by many countries [1]. Although underground mining has some advantages in the management of the empty areas of mines, it causes serious damage to soil ecosystems, leading to the surface subsidence and degradation of vegetation, resulting in the reduction of biodiversity and the stability of ecosystem functions [2,3]. Therefore, the environmental management and ecological restoration of underground mining areas is a top priority and has attracted much attention.

For decades, there have been efforts to restore ecosystems damaged by mining activities. Vegetation restoration was considered to be an effective way of ecological environment management in mining areas and an essential indicator of ecological environment restoration in mines [4]. Nature-based solutions are the preferred strategy for mine revegetation due to their cost-effectiveness and absence of secondary pollution [5]. However, heavily compacted and poorly fertilized mine soils lack essential nutrients such as carbon and nitrogen, severely limiting the nutrient requirements for vegetation restoration [4]. This may slow the natural recovery process. Therefore, it is important to study soil biochemical cycles in mining areas for vegetation recovery.

As the most widely distributed and active components of surface ecosystems, microorganisms are important in driving processes such as ecosystem material cycling and nutrient transformation, and are regulators of subsurface ecological processes [3,6]. Microorganisms can help restore ecosystem function and play an important role in mine restoration [7]. Microorganisms dominate a series of important soil functions by regulating nutrient cycling, improving soil structure, decomposing organic matter, and suppressing soil- transmitted plant diseases [8]. Xu et al. [7] found that microbial communities were strong drivers of soil multifunctionality. Bacterial diversity was positively and non-redundantly correlated with soil carbon cycle, nitrogen cycle function, and soil multifunctionality [7]. Mining activities cause serious destruction of soil ecosystems and have a deleterious effect on the soil microbiome. It was found that mining significantly decreased microbial diversity, richness and microbial biomass [9]. And as the restoration years increase, the microbial diversity gradually recovers, but the consequences of mining still exist [7,9]. The impact of mining activity on microorganisms is not only on the bacterial community, but also on the composition of the mycorrhizal fungal community, which has been altered and has not recovered after 11 years of reclamation [10]. Guan et al. [11] found that the composition of the soil microbial community changed significantly with the gradient of distance from the mine site. Soils closer to the mine are more disturbed, with unstable soil structure and severe nutrient loss, than areas outside the undisturbed mine [12]. Disturbance from mining activities at mines diminishes as distance from mines increases [12].

Rhizosphere soil microbial community has been demonstrated to promote plant community restoration by influencing ecosystem functions such as element cycling and primary production [6]. If only revegetation, terrain modification, soil improvement and other projects are implemented, and the participation of microorganisms is neglected, the potential for ecological restoration is limited, and the effect and rate of restoration are ineffective [13]. Therefore, ecological restoration of mines must emphasize soil microbial succession, ecological processes and their recovery potentials [14]. Yang et al. [3] found that microbial community α-diversity was significantly higher (both fungal and bacterial community) and the microbial network was more stable in the vegetation restoration area than in the non-restoration area. Ma et al. [4] found that vegetation type significantly affected the structural composition of soil rich and rare microbial communities, and different vegetation restoration types increased the network topological parameters and complexity of both rich and rare microbial communities. These studies suggest that vegetation restoration processes are closely associated with below-ground microbial communities. Microorganisms are also an important indicator of ecological restoration in mining areas [15,16]. Microorganisms are an important tool for soil quality enhancement and biodiversity restoration in mines, and understanding the relationship between important functional groups of microorganisms and soil factors is crucial for ecological restoration in mines.

Previous studies on bacterial communities have focused on the effects of plants on bacterial communities in mine reclamation areas [3–4], while ignoring the effects of the mining process. Bacterial and fungal communities affected by opencast coal mining at distances of 1500 and 1000 meters [17]. The impact of underground mining on bacterial communities remains unclear. It is also not clear whether rich and rare taxa respond consistently to disturbance. Rich taxa have wider ecological niches, their community assembly processes are usually dominated by stochastic processes, and they have a greater responsibility in maintaining the community structure stability [18]. Therefore, in this study, we selected the soil of Shangwan coal mine as the research object and set different distances from the mine to study the effects of mine disturbance on the α-diversity, β-diversity, bacterial community composition, and bacterial community assembly model of the soil bacterial community, to reveal the impacts and mechanisms of mine mining on the inter-root bacterial community of plants, and to provide new insights for the natural restoration of damaged mine ecosystems.

## 2. Materials and methods

### 2.1. Study area

**Fig 1:**
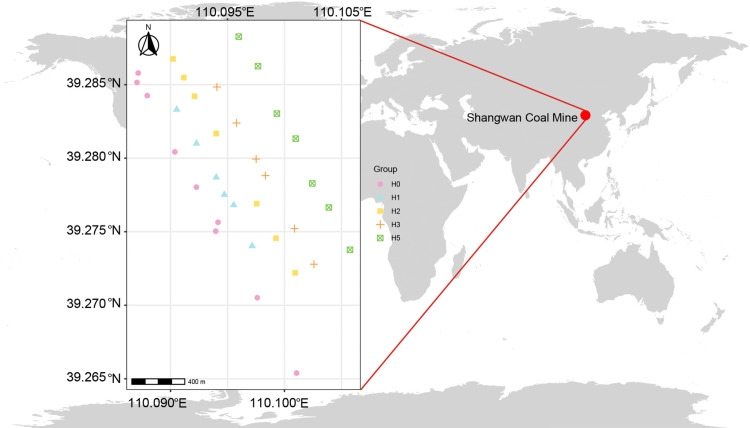
Location of study area and sampling sites. The world map came from the maps package, with the most original source being Natural Earth (https://www.naturalearthdata.com). The dots were sampling locations.

Shangwan Coal Mine is located in Yijinholo Banner, Ordos City, Inner Mongolia Autonomous Region (E109°30′-110°30′, N38°50’-39°50′. Fig 1). The study area has a temperate continental climate, with an average annual temperature of 7.4 °C and an average annual rainfall of 437.5 mm [19]. The average annual number of days of rainfall is 67.8 d, which is generally concentrated in July-August [19]. The soil is mainly sandy soil [19]. The vegetation type belongs to the transition from steppe to desert steppe and consists of perennial, dry and tufted grasses with dry shrubs and semi shrubs. The dominant species are mainly *Caraganaintermedia*, *Thymusmongolicus*, *Setariaviridis*, *Cleistogenesramiflora* and *Lespedeza bicolor Turcz*, *Caragana Korshinskii Kom.*, etc.

### 2.2. Soil sampling

Along the vertical direction of the underground mine tunnel, we set multiple distances for soil collection. Due to underground coal mining, we considered the intensity of disturbance to be reduced compared to open pit coal mining, so we set the maximum distance to be half the distance of the impact of open pit coal mining (1500 m) on the bacterial community [17]. We selected 5 distances at 0 m (H0), 150m (H1), 300m (H2), 450m (H3) and 750m (H5) to collect rhizosphere soil (Fig 1). The 10m × 10m large sample square was set up and three 1m × 1m small sample squares were set up within the large sample square by diagonal method. Soil samples were collected within the three small sample squares and mixed to be considered as one sample. Since it was not convenient to unfold large 10m × 10m samples at the H0 distance, we chose three closer samples to mix the soil to be considered as one sample. The soil 1–2 cm distance from the roots was identified as rhizosphere soil. A minimum of 6 replicates were set for each distance. Due to the low vegetation cover in H0 distance, 9 samples were collected to minimize the error. The soil was divided into two parts, one for the determination of soil physicochemical properties, and the other was stored at -80 °C for 16s rRNA sequencing.

### 2.3. Determination of soil physical and chemical properties and microbial sequencing

Soil pH and salinity were measured by potentiometric method [20]. SOM was determined by potassium dichromate heating method [20]. TN content was determined using an elemental analyzer [20]. SUE was determined by sodium phenol-sodium hypochlorite colorimetric method [4]. SALP was determined by sodium benzene diphosphate colorimetric method [4]. the SSC activity was determined by DNS colorimetric method [4]. Soil TP was determined using the sulfuric acid-perchloric acid digestion-colorimetric method [14]. Soil TK was determined using the flame photometer method [14].

16s rRNA sequencing was done by biotechnology company (Guangdong Magigene Biotechnology Co., Ltd. Guangzhou, China).After genomic DNA extraction using the DNA extraction kit ((E.Z.N.A.® Soil DNA Kit, Omega Bio-tek), concentration of DNA were detected by 1% agarose gel electrophoresis, and the samples were diluted to 1 ng/μL with sterile water in a centrifuge tube. The V4-V5 region of the bacterial 16S rRNA gene was amplified with specific primers 515F (5’-GTGCCAGCMGCCGCGGTAA-3’) and 907R (5’- CCGTCAATTCMTTTRAGTTT-3’). The products were detected by 2% agarose gel electrophoresis, and purified by using AxyPrep DNA Gel Extraction Kit. The constructed libraries were sequenced using an Illumina Nova6000 platform. Trim reads with average quality scores (Q-score) < 20 over a sliding window. DADA2 algorithm was used to denoise. De-redundancy was not used. Annotation was finished by the SILVA database.

### 2.4. Data analysis

All data processing was done through R (4.2.1) software. OTUs with relative abundance greater than 0.1% (min) and less than 0.01% (max) per distance were defined as abundant and rare taxa. Since it was each distance that was categorized, the OTUs in the abundant and rare taxa differed across multiple distances. Therefore, when combining different distances, if the OTUs of a single distance are defined as abundant taxa, the actual value is retained, whereas a value of 0 is assigned if it is not defined as an abundant taxon by other distances. So far, an OTUs abundance table of abundant and rare taxa was obtained. The vegan package [21] was used to calculate α-diversity and β-diversity of the bacterial community. ANOVA and LSD multiple comparisons were performed through the agricolae package [22]. Transformation of data with skewed distributions followed by ANOVA. Bacterial community phylum level chords were plotted by circlize package [23]. Multiple comparisons of bacterial composition were performed by the microeco package [24]. Multiple regression of microbial diversity indices with soil environmental factors was performed and the contribution of environmental factors was calculated by relaimpo package [25]. In addition, the relationship between bacterial community diversity and soil environmental factors was determined by univariate linear regression.

The molecular ecological network was analyzed using the WGCNA [26] and igraph packages [27]. The top 250 OTUs with high relative abundance were selected for analysis in the molecular ecological network. Interactions between bacterial OTUs were characterized by spearman correlation. Relationships with absolute value of correlation coefficients >0.8 and p < 0.01 were considered as OTUs being interacting. Visualization of the network using Gephi software (0.9.2). The network topology properties are described by calculating the average clustering coefficient, average path distance, network density and modularity, and the role of each node in the topology is determined based on 2 attributes: intra-module connectivity (Zi) and inter-module connectivity (Pi). Based on previous studies [28], all nodes were categorized into four subclasses: peripheral nodes (Zi < 2.5, Pi < 0.62), modular hubs (Zi > 2.5, Pi < 0.62), connecting nodes (Zi < 2.5, Pi > 0.62) and network hubs (Zi > 2.5, Pi > 0.62).

Null modeling [29] was used to analyze microbial community ecological processes and explore microbial community assembly processes on the Cloud online platform (www.majorbio.com). Parameters such as β-nearest taxon index (βNTI) and Raup-Crick matrix (RCbray) are first calculated and obtained. When |βNTI|>2, it indicates that the community assembly process is deterministic, where βNTI > 2 indicates heterogeneous selection and βNTI < -2 indicates homogeneous selection. When |βNTI|<2, it indicates that community assembly is dominated by a stochastic process, where RCbray>+0.95, RCbray < -0.95 and | RCbray|<0.95 indicate that the stochastic process is dispersal limited, homogeneous dispersal and undominated process, respectively.

## 3. Results

### 3.1. The α-diversity and NMDS analysis of bacterial community

**Fig 2:**
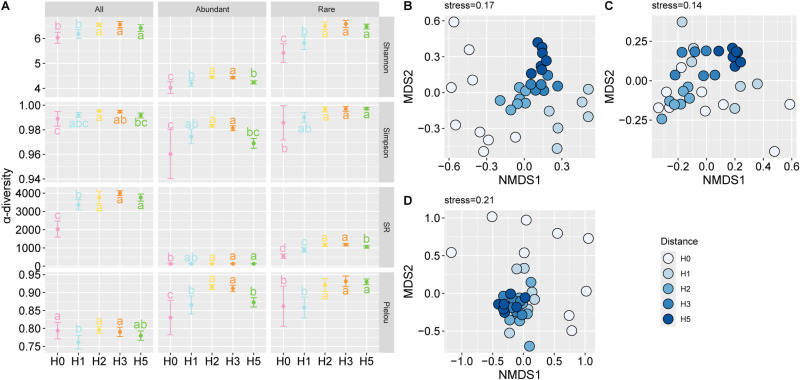
Theα-diversity (A) and NMDS analysis of bacterial community of total (B), rich (C) and rare (D) taxa. SR, species richness.

The shannon index and richness index of the total bacterial community increased (Fig 2A, P < 0.05) with distance from the mining area and reached a maximum at distance H5, while the simpson index showed a trend of increasing and then decreasing, and then reached a maximum at distance H2 (P < 0.05), and was higher than that of H0, although it decreased at distances H3 and H5. There was an increasing trend in rare α-diversity indices with increasing distance from the mining area. In contrast, the α-diversity of rich species tended to increase and then decrease, and reached a maximum at the H2 distance.

NMDS analysis showed significant changes in the bacterial community structure (Fig 2). In total, the bacterial community structure of H0 was significantly different from that of the other distances (Fig 2B). The total bacterial communities at H2, H3, and H5 showed significant clustering, indicating that the overall bacterial community structure was similar among the three distances. In terms of rich species, the 5 distances produced an insignificant distance in terms of community structure (Fig 2C). In rare species, the bacterial community structure was similar at all distances except for H0 in the mining area (Fig 2D). In addition, we found that there was strong heterogeneity in the community structure in H0. PMANOVA analysis showed significant effects of distance on bacterial community structure for overall, abundant and rare taxa.

### 3.2. Community composition analysis

**Fig 3:**
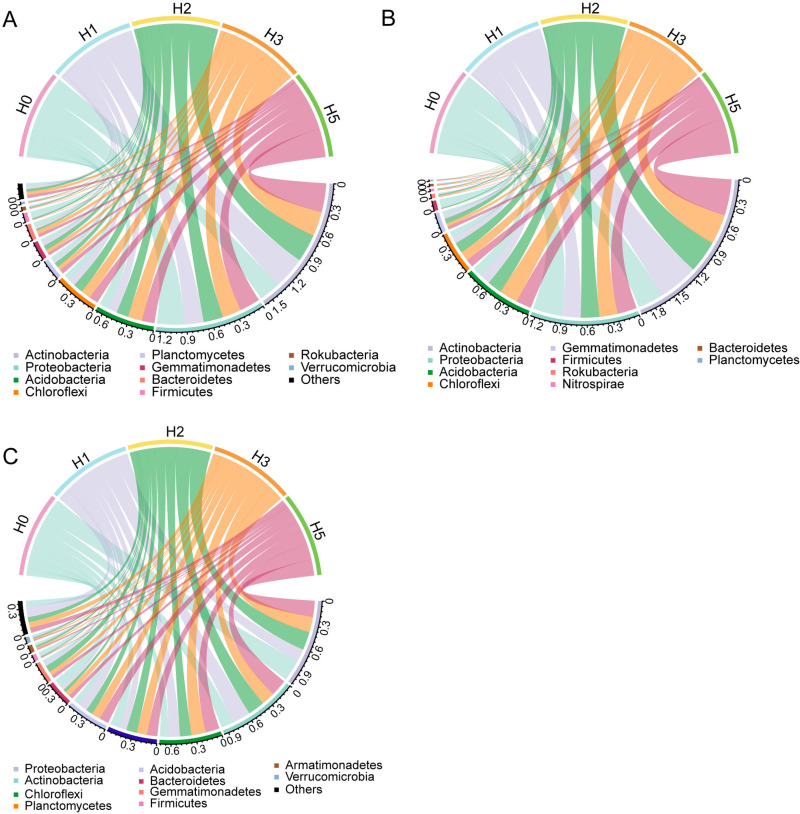
The relative abundance of bacterial community at phylum. A, total taxa. B, rich taxa. C, rare taxa.

Both Actinobacteria and Proteobacteria had high relative abundance in the total, abundant and rare bacterial community (Fig 3). It was found that in total (Fig 3A), compared to H0, the other distances increased the relative abundance of Actinobacteria, Acidobacteria, and Chloroflexi, which reached the maximum at H1, H3, and H3, respectively, but significantly decreased the relative abundance of Proteobacteria and Firmicutes. Planctomycetes had high relative abundance in the rich bacterial community (Fig 3B), and the four phyla (Actinobacteria, Proteobacteria, Acidobacteria, Chloroflexi) with higher relative abundance varied with distance similarly to the total. In the rare bacterial community (Fig 3C), Proteobacteria had the highest relative abundance, followed by Actinobacteria, Chloroflexi, Planctomycetes, Acidobacteria and Bacteroidetes. In contrast to the total and rich bacterial community, the Bacteroidetes varied considerably at different distances, and distance expansion reduced the relative abundance of the rare Bacteroidetes. We performed a random forest analysis (S1 Fig) and found that the Firmicutes had high relative abundance in the H0 treatment, whether in total, abundant or rare taxa, and the Acidobacteria had higher relative abundance at H3. The relative abundance of Bacteroidetes varied across taxa, with the highest relative abundance at H3, H2 and H5 distances, respectively.

**Fig 4:**
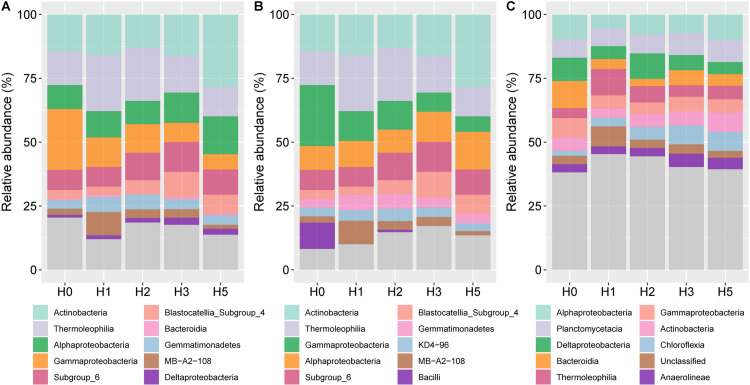
The relative abundance of bacterial community at class. A, total taxa. B, rich taxa. C, rare taxa.

In class level, both the total, rich and rare taxa had the highest relative abundance of the Alphaproteobacteria (Fig 4). The relative abundance of Actinbacteria and Alphaproteobacteria of total taxa was significantly higher in the H5 distance than in the other distances (Fig 4A). The relative abundance of Gammaproteobacteria decreased gradually with increasing distance, while subgroup_4 and subgroup_6 increased. The relative abundance of Thermoleophilia increased and then decreased with increasing distance, reaching maximum and minimum values at H1 and H5, respectively. In the rich taxa (Fig 4B), the variation in bacterial phyla was similar to that in total. The difference was that Bacilli had higher abundance and was significantly higher at the H0 distance than the other distances. Among the rare taxa (Fig 4C), Planctomycetacia had higher relative abundance. Chloroflexia increased significantly with increasing distance, and the relative abundance of Anearolineae was significantly higher at the longer distances (H3, H5) than at the closer distances.

### 3.3. Network analysis

**Fig 5:**
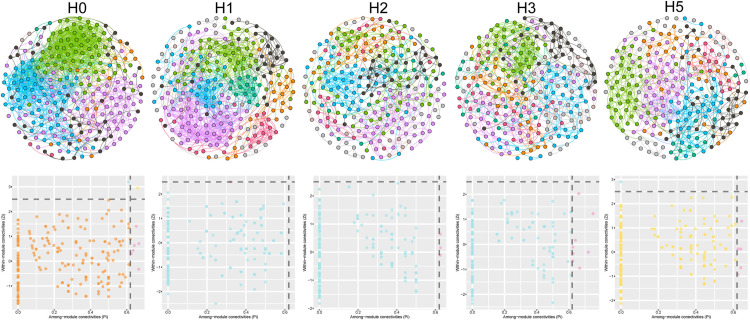
The network analysis and Zi-Pi analysis of bacterial community at different distances.

The number of nodes in the symbiotic network is generally similar for different distances (Fig 5, Table 1), but the number of edges differs significantly, with increasing distance to reduce the number of edges in the network. The H0 distance has the smallest average path length and the highest network density, indicating that the network at H0 is more connected. A module hubs existed in H0 only. To further characterize the resistance (robustness) of microbial networks to interference, the magnitude of altering natural connectivity by removing nodes was tested (S2 Fig), and the results showed that after removing the same percentage of nodes, the stability of all symbiotic networks decreased with the percentage of removal. H0 possessed the highest stability, while H5 had the lowest stability, indicating a gradual decrease in the stability of the bacterial community network with increasing distance from the mine.

**Table 1:** The network parameter at different distances.

	nodes	edges	Positive edges	Negative edges	Average degree	Average path length	Network diameter	Network density	Clustering coefficient
H0	241	1299	735	564	10.7801	3.8889	12	0.0449	0.5222
H1	240	1104	584	520	9.2000	5.1753	14	0.0385	0.5311
H2	240	567	417	150	4.7250	6.1269	15	0.0198	0.4764
H3	240	675	442	233	5.6250	6.4792	17	0.0235	0.5304
H5	237	653	351	302	5.5105	5.3874	14	0.0233	0.4437

### 3.4. Community assembly processes analysis

**Fig 6:**
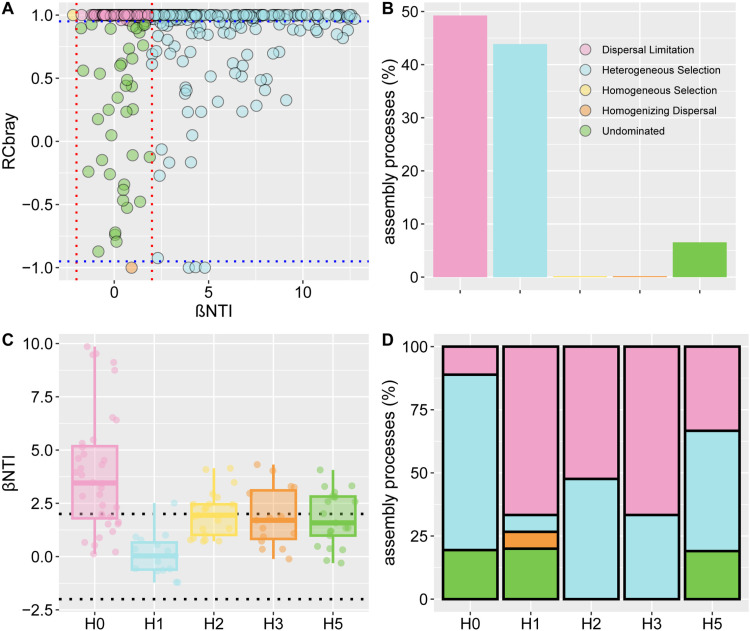
Community assembly processes analysis. A and B, the βNTI and RCbray distribution and contribution of assembly processes among all distances. C, the βNTI of distribution in different distances. D, the contribution of assembly processes different distances.

Dispersal limitation contributed the most to bacterial community construction with 49.24%, followed by heterogeneous selection with 43.87% and nondominant contribution with 6.55% (Fig 6A, B). The βNTI of H0 was higher than 2, indicating that H0 was dominated by deterministic processes (Fig 6C). The deterministic process of H0 was mainly from heterogeneous selection with a contribution of 69.4% (Fig 6D). The community assembly process in H1 was dominated by stochasticity, with a deterministic contribution of only 6.67%. H2, H3, and H5 distances have similar stochastic and deterministic contributions, with stochastic contributions all higher than deterministic contributions. And the stochastic contribution source is mainly dispersal limitation.

### 3.5. Effects of environmental factors on bacterial community

**Fig 7:**
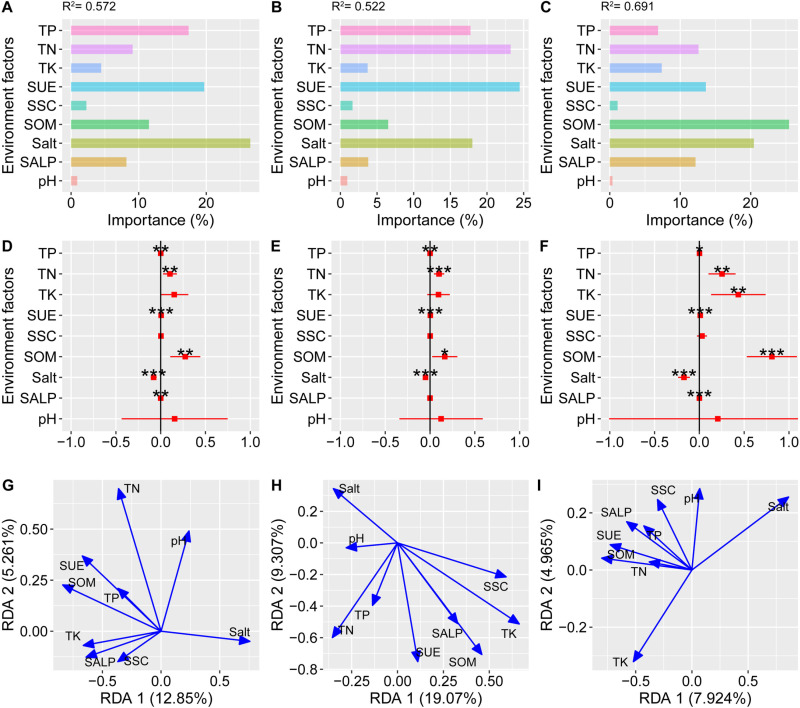
The relationship between environmental factor and bacterial community the α-diversity and structure. A, B and C, the importance of environmental factors to Shannon index of total, rich and rare taxa in multiple regression. D, E and F, the estimation of environmental factors to Shannon index of total, rich and rare taxa in univariate linear regression. G, H and I, the RDA analysis of total, rich and rare taxa.

We examined the contribution of environmental factors to the shannon index of bacterial community by multivariate regression analysis and found that 9 soil environmental factors explained 57.2%, 52.2% and 69.1% of the variation in the diversity of total, rich and rare taxa, respectively (Fig 7A, B, C). Among total taxa, soil salt, SUE and TP had the highest contribution to the α-diversity variation with 26.5%, 19.7%, and 17.4%, respectively (Table 2). Among the rich taxa, SUE, TN, TP, and salt contributed 24.4%, 23.2%, 17.7%, and 17.9%, respectively, to the shannon index. SOM has the highest contribution, explaining 25.4% of the rare taxon. There was a significant positive correlation between bacterial community α-diversity and TP, TN, SUE, SOM diversity and a significant negative correlation with salt (Fig 7D, E, F). A significant positive correlation was found between SALP and the shannon index in total and rare bacterial taxa. The two RDA axes explained 18.11%, 28.38% and 12.89% of the variation in the bacterial community structure of the total, rich and rare taxa (Fig 7G, H, I). Monte Carlo test showed that TK, SOM, SUE and SALP significantly affected the bacterial community structure of the 3 taxa. TN had a significant effect on total and rich bacterial community structure, explaining 43.9% and 17.3% of the variation, respectively. The salt affected total and rare taxa community structure (R^2^ = 0.503, 0.642). pH and SSC significantly affected total and rich taxa community structure (R^2^ = 0.206, 0.255), respectively. SOM had the greatest effect on to total bacterial community structure (R^2^ = 0.651). TK and salt had the greatest effect on the community structure of rich and rare taxa, respectively (R^2^ = 0.483, 0.642).

**Table 2:** Monte Carlo test of total, rich and rare taxa. The ***, ** and * indicate that the R^2^ is significant at 0.001, 0.01 and 0.05 level.

Environment factors	Total	Rich	Rare
TN	0.439***	0.173*	0.113
TP	0.148	0.069	0.159
TK	0.386***	0.483***	0.301**
Salt	0.503***	0.165	0.642***
SOM	0.651***	0.471***	0.505***
SUE	0.487***	0.314***	0.418***
SALP	0.367**	0.244*	0.282**
SSC	0.133	0.255*	0.118
pH	0.206*	0.040	0.068

**Fig 8:**
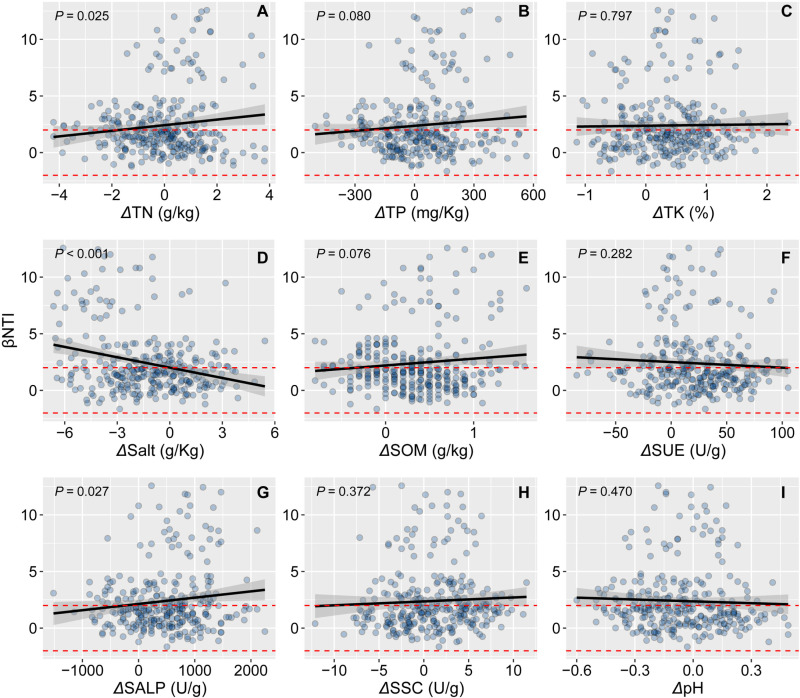
The relationship between the βNTI and differences of environmental factors.

The relationship between βNTI and environmental factors was used to infer changes in the relative contribution of deterministic and stochastic assembly processes (Fig 8). TN, salt, and ALSP significantly predicted βNTI. βNTI increased significantly with increasing differences in TN and ALSP and decreased with increasing salt differences. This suggests that increased salt differences changed the bacterial community composition from deterministic to stochastic. In contrast, the differential increase in TN and ALSP shifted bacterial community assembly from stochastic to deterministic.

## 4. Discussion

### 4.1. Effects of mine disturbance on bacterial community diversity and structure

In this study, it was found that the diversity of both total, rich and rare bacterial community increased with increasing distance from the mine (Fig 2). More species were found from the mine site outwards, which is consistent with previous results [30]. Mining activities have altered soil factors and some soil biogeochemical processes, thereby affecting soil microbial community in terrestrial ecosystems [1]. Song et al. [19] found a significant decrease in vegetation cover in the Shangwan mining area as a result of mining. Our investigation found that plant diversity and cover decreased with increasing distance from the mine. Due to the frequent disturbance of the soil in mining areas, plant growth is unstable and poorly adapted, microorganisms activity are decreased. Plant diversity is a critical factor influencing bacterial community, and diversified plant root exudates may support more microbial taxa by increasing resource availability and altering physical microhabitat and environmental conditions [31]. Vegetation selectively attracts specific microorganisms through direct and indirect effects, resulting in the enrichment of such microorganisms in soil, leading to changes in microbial diversity [32]. Overall, we concluded that it was the mining activities that first interfered with plant (diversity and coverage), which in turn affected the diversity of the microbial community.

We found that SOM is a powerful predictor of changes in microbial diversity. The removal or damage of surface vegetation and the destruction of the original stratigraphic structure during mining, resulting in the destruction of soil aggregates and the decomposition of SOM [33,34]. On the other hand, the natural topsoil is unregulated stripping, disordered mixed discharge, lack of natural topsoil cover mining SOM content is extremely low [34,35]. In this study, H0 was sparsely vegetated with low cover. This can lead to long-term wind erosion, resulting in low soil nutrient and SOM content [36].

In terms of β-diversity, mine disturbance had a significant effect. There were large differences within the H0 group, suggesting that mine disturbance led to changes in the structure of the bacterial community. The NMDS showed that sampling distance had a significant effect on the total and rare taxa bacterial community structure, while it had a smaller effect on the rich taxa bacterial community structure, suggesting that mining alters the bacterial community structure mainly by changing the rare species. Rich species typically occupy a wider ecological niche width in the ecosystem and can utilize more resources, which makes them more resilient to external environmental perturbations than rare species [37]. RDA showed that salt, SOM and SUE were the main soil factors affecting rare bacterial community structure. The salt is an important factor that affects the bacterial community [38]. Under high salt, rich species are more stable to changes in salt, while rare species are more sensitive [39]. SUE and other enzymes are closely related to soil N mineralization and cycling, and are able to characterize the N nutritional condition of the soil. Ma et al. [4] found that SUE had a significant effect on the structure of soil bacterial community in the mining area, whereas there was a positive relationship between the soil enzyme activity content and plant diversity. In this study, the gradual improvement of soil nutrients with increasing distance contributed to an increase in the activity of microorganisms involved in the N cycle, which led to an increase in SUE activity in response to nutrient depletion.

### 4.2. Effects of mine disturbance on bacterial community composition

This study showed that Actinobacteria, Proteobacteria, Chloroflexi, and Acidobacteria were the dominant abundant bacterial phyla in the soil of this mining area, which is in consistent with previous studies [4,32,40]. This may be due to the fact that these phyla are better adapted to the environment and have high ecological niches. Actinobacteria are the main decomposers of cellulose and lignin in the soil and can survive better in poor soils. Liu et al. [41] found that the relative abundance of the bacterial Actinobacteria increased significantly with the increase of plant litter, and the Actinobacteria showed a significant positive correlation with SOM. With increasing distance from the mine, plant cover increased, which resulted in more abundant root secretions and plant litter in the soil, increasing the relative abundance of the Actinobacteria. It was found that increasing distance decreased the relative abundance of Proteobacteria. Proteobacteria can adapt to high-salt environments [20], and there are a large number of N-fixing bacteria in Proteobacteria [42,43], which are able to survive well in N-poor soils. The Chloroflexi phylum is usually rare in rhizosphere soil, but in this study, it consistently had high relative abundance. Sun et al. [44] reported that the Chloroflexi can be used as a marker of mine restoration and can help plant colonization and growth. In the present study, it was found that the relative abundance of the Firmicutes was significantly higher at H0 in both rich and rare taxa. Previous studies have found that the Firmicutes is the most dominant phylum during ecological restoration, while its relative abundance decreases significantly during the middle and late stages of restoration, which is consistent with our results [45]. The relative abundance of Gammaproteobacteria is often negatively correlated with plant establishment, and Gammaproteobacteria contain diazotrophic taxa with antagonistic interactions or competition for limited available nutrients at the species or ecotype level [44,46], which is consistent with our results. Bacilli is a plant rhizosphere-promoting bacteria that mitigates the stressful effects of the environment on plants [47]. The abundance of Bacilli at higher H0 distances again indicated that plants in this region are exposed to great stress. This pressure may be due to a variety of factors, such as heavy metals, groundwater pollution, surface cracking, and many other factors.

### 4.3. Effects of mine disturbance on networks

Molecular ecological network analysis can reveal soil microbial species interactions. In this study, OTUs with high relative abundance were selected for the construction of networks. The results showed that there was no significant difference in the number of nodes at different distances, but the number of edges and the average path length of the molecular ecological network gradually decreased and increased as the distance increased. The H0 distance has the highest robustness by randomly removing nodes. This suggests that soil bacterial community in the mining area has a higher disturbance tolerance with close communication and ability to respond to mining disturbances accordingly. The complexity and stability of molecular ecological networks were mainly controlled by core species [48]. Peng et al. [49] found that drought treatment decreased microbial α-diversity but increased the complexity and stability of molecular ecological networks. Li et al. [50] also found that as soil depth increased, nutrient depletion increased, microbial diversity decreased, and the strength of interactions between microorganisms increased, which is consistent with our results.

Molecular ecological network analysis also facilitates the exploration of key taxa, which play important roles in the microbiome and whose absence or absence leads to significant changes in microbial structure and function. In this study, we searched for key taxa of microbial community through Zi and Pi. The results showed that only one OTU (Class: Phycisphaerae) existed in H0 treatment belonging to Network hubs. Previous studies have found a significant negative correlation between the relative abundance of Phycisphaerae and plant growth. The importance of Phycisphaerae in the ecological network gradually decreased with increasing distance and vegetation cover. Ma et al. [14] compared the molecular ecological networks of soil microbial community in different mining areas and found that there were no Network hubs in the network. Yang et al. [3] studied the molecular ecological network of rhizosphere soil bacterial communities of different vegetation in the mining area and found that there were no Network hubs, which is consistent with our results.

### 4.4. Effects of mine disturbance on bacterial community assembly processes

Deterministic and stochastic processes co-regulate community assembly, but their relative importance differs across environments. This study showed that soil bacterial communities at H0 distance had |βNTI|>2, indicating that deterministic processes dominated bacterial community construction, and | RCbray|>0.95 suggesting that deterministic processes was the main contribution from heterogeneous selection. Yin et al. [51] studied the soil bacterial community assembly process in different restoration areas of the mine and found that the bacterial community assembly process in the natural restoration area, the restored area and the disturbed area were dominated by stochasticity, while the tailings area was mainly dominated by deterministic processes, which is consistent with our results. Deterministic processes have been shown to be better than stochastic processes in environments with high selection intensity. This means that bacterial communities can respond more quickly to environmental changes and may be more susceptible to deterministic processes such as environmental filtering or biological interactions [52]. As the external environment becomes harsh, deterministic processes dominate community assembly processes. The H0 distance is more disturbed by mining and the plant and soil environment is unstable, so the filtering of the environment leads to a higher deterministic processes. This further indicated that the environment at H0 distance is harsh for soil bacterial communities. while other distances were dominated by stochastic processes, suggesting that distance mitigates the effects of mining disturbance on bacterial communities.

## 5. Conclusion

In this study, we explored the effect of disturbance on bacterial communities by studying the bacterial communities responding to the distance from the mine. The results showed that mining had a significant effect on the α-diversity and β-diversity of the bacterial community, with more significant effects on rare taxa. In terms of β-diversity, the response of abundant taxa to mine disturbance was not significant, while the effect of rare taxa was significant. Thus, mine disturbance has mainly affected the structure of rare taxa. The poor soil and low vegetation cover in the mining area makes the bacteria more connected to each other and the network more stable. Disturbed by mining, the bacterial community assembly process in the mining area was dominated by deterministic selection. As distance increases and disturbance decreases, bacterial community assembly is dominated by stochasticity. Mining disturbances have led to environmental changes that affect the assembly of bacterial communities. Salt is the most key factor affecting the bacterial community assembly process and at the same time, salt affects the overall structure by influencing the structure of the rare taxa. This study provides a new approach for the ecological restoration of fragile mining ecosystems. In the future, we will further strengthen the study of functional networks and enrich the understanding of self-restoration mechanisms.

## Supporting Information

S1 Fig The random forest analysis of bacterial community at phylum. A, total taxa. B, rich taxa. C, rare taxa.
(TIF)

S2 Fig Targeted removal robustness analysis. (TIF)
