# Supplementary figures and images for: Effects of underground coal mining disturbance on bacterial community diversity and its assembly processes
Source: PLoS One. 2025 May 7;20(5):e0322014. doi: 10.1371/journal.pone.0322014 (PMC12057849; doi:10.1371/journal.pone.0322014)

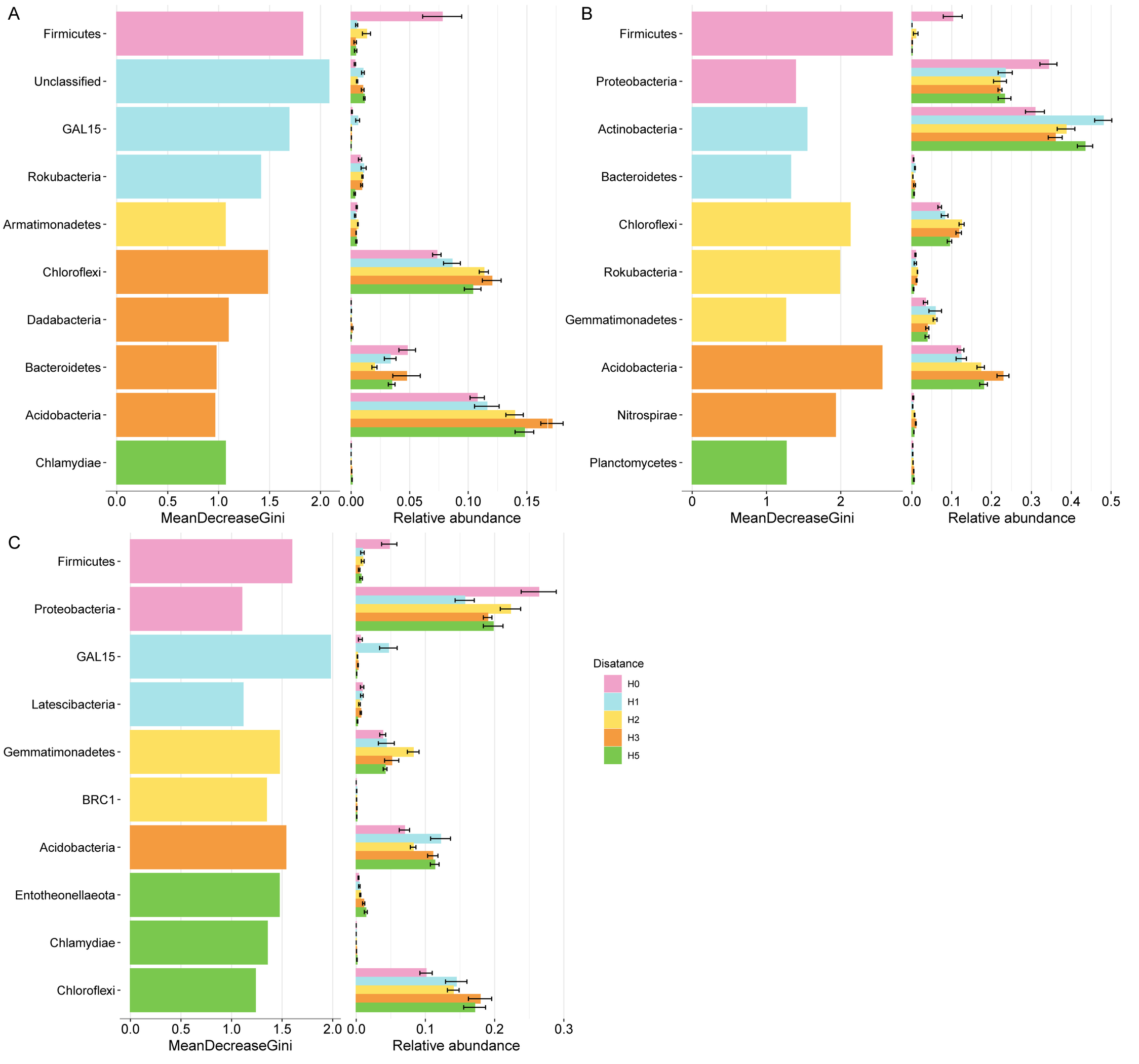

Supplement: S1 Fig — A, total taxa. B, rich taxa. C, rare taxa. (TIF) [file pone.0322014.s001.tif]
